# The rainfall plot: its motivation, characteristics and pitfalls

**DOI:** 10.1186/s12859-017-1679-8

**Published:** 2017-05-18

**Authors:** Diana Domanska, Daniel Vodák, Christin Lund-Andersen, Stefania Salvatore, Eivind Hovig, Geir Kjetil Sandve

**Affiliations:** 1 Department of Informatics, University of Oslo, Oslo, Norway; 2 Statistics For Innovation, Norwegian Computing Center, Oslo, Norway; 3 Institute for Medical Informatics, The Norwegian Radium Hospital, Oslo University Hospital, Oslo, Norway; 4 Department of Tumor Biology, Institute for Cancer Research, Oslo University Hospital, Oslo, Norway

**Keywords:** Rainfall plot, Visualization, Mutation, Genomics

## Abstract

**Background:**

A visualization referred to as rainfall plot has recently gained popularity in genome data analysis. The plot is mostly used for illustrating the distribution of somatic cancer mutations along a reference genome, typically aiming to identify mutation hotspots. In general terms, the rainfall plot can be seen as a scatter plot showing the location of events on the x-axis versus the distance between consecutive events on the y-axis. Despite its frequent use, the motivation for applying this particular visualization and the appropriateness of its usage have never been critically addressed in detail.

**Results:**

We show that the rainfall plot allows visual detection even for events occurring at high frequency over very short distances. In addition, event clustering at multiple scales may be detected as distinct horizontal bands in rainfall plots. At the same time, due to the limited size of standard figures, rainfall plots might suffer from inability to distinguish overlapping events, especially when multiple datasets are plotted in the same figure. We demonstrate the consequences of plot congestion, which results in obscured visual data interpretations.

**Conclusions:**

This work provides the first comprehensive survey of the characteristics and proper usage of rainfall plots. We find that the rainfall plot is able to convey a large amount of information without any need for parameterization or tuning. However, we also demonstrate how plot congestion and the use of a logarithmic y-axis may result in obscured visual data interpretations. To aid the productive utilization of rainfall plots, we demonstrate their characteristics and potential pitfalls using both simulated and real data, and provide a set of practical guidelines for their proper interpretation and usage.

**Electronic supplementary material:**

The online version of this article (doi:10.1186/s12859-017-1679-8) contains supplementary material, which is available to authorized users.

## Background

The rainfall plot (RP) can be seen as a scatter plot showing the location of events on the x-axis versus the distance to their respective preceding event on the y-axis.

The plot is mostly used for detecting mutation hotspots in cancer genomics by visualizing the distribution of somatic point mutations (SPMs) along a reference genome. In this case, each event is a mutation. The x-coordinate shows the genomic position of the mutation, while the y-coordinate represents the base pair distance between consecutive mutations on a logarithmic scale.

To our knowledge, the RP was first used to visualize SPMs in a paper by Nik-Zainal et al. in 2012 [1]. It has since been widely used for studying patterns of genomic mutations (e.g., [1–5]).

However, the interpretation of the RP is not fully intuitive, and several challenges need to be overcome to allow its productive use. The first challenge is to correctly read out the density of mutations in the various genomic regions of interest.

The second challenge is to take into account potential congestion in the plot, i.e. that multiple mutations share the same x-y-coordinate and thus appear as a single mutation.

A third and related challenge concerns the usage of multiple colors for highlighting subsets of the displayed data. The (in principle arbitrary) order in which the mutation subsets are plotted may affect the resulting color at a given x-y-coordinate due to congestion. The plotting order may in this way strongly affect the visual impression of which subsets are the most frequent.

We here aim to guide researchers in correctly utilizing and interpreting RPs by explaining its characteristics and pitfalls. Our conclusions are based on a careful inspection of RP properties and illustrated using real mutation data. We also critically evaluate when the RP may be the best means of visualizing mutational patterns along a genome, and when a visualization like a traditional frequency plot would be preferable.

## Results and discussion

We first provide a formal definition of the RP, and then present the challenges of visualizing mutations of a large (e.g. human) genome. We discuss how RPs offer a partial solution to some of these challenges, what the limitations of the RP are, as well as some particular caveats that should be kept in mind when creating and interpreting an RP of mutation data.

### Formal definition of rainfall plot

We define the *RP* for a strictly monotonically increasing sequence of integers (*p*_1_,…,*p*_*N*_) as a scatterplot of the points *S*={(*x*_*i*_,*y*_*i*_) | *i*∈{1,2,…,*N*−1}} where each *y*_*i*_ is given by:

$$\begin{array}{*{20}l} y_{i} &= \log(p_{i+1}-p_{i}) \quad \forall \, i \in \{1,2,\ldots, N-1 \} \end{array} $$

and each *x*_*i*_ is given by:

$$\begin{array}{*{20}l} x_{i} &= p_{i+1} \quad \forall \, i \in \{1,2,\ldots, N-1 \} \end{array} $$

When making an RP for mutations within a single chromosome, the chromosome offsets of mutations can be used directly in the above formula. When making an RP for a whole genome, mutations across chromosomes need to be combined into a single plot. Also, in order to fit a grid of a specific size (to have full control of how a plot will be displayed on a screen or printed on paper), the x- and y-values need to be scaled in accordance with the grid size. Full details are provided in Additional file 1.

### Visualizing the distribution of mutations along a genome

Human SPMs are determined as individual genomic positions having different alleles in somatic cells compared to the germline of a particular individual. SPMs can be represented as a set of mutations occurring at particular point locations along the ∼3 billion base pairs of a human reference genome.

**Fig. 1:**
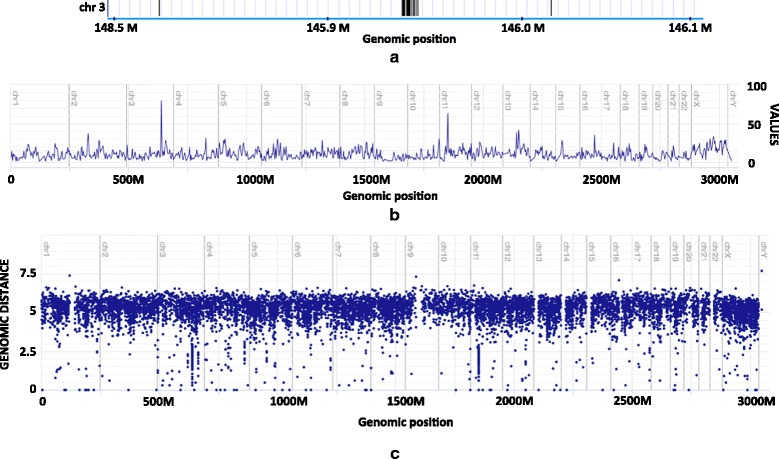
Visualization of SPMs in a pancreatic cancer patient. SPMs (marked with *vertical black lines*) at location 145,800,000−146,100,000 of chromosome 3 of the hg19 human reference genome (**a**). A frequency line plot (**b**) and RP (**c**) showing kataegis regions at location ∼150 Mbp in chromosome 3 (corresponding to whole-genome location ∼650 Mbp in the figure) and at location ∼35 Mbp in chromosome 11 (corresponding to whole-genome location ∼1850 Mbp in the figure). Data taken from [2]

Figure 1 a shows a ∼20 kbp long region of chromosome 3 enriched with SPMs in the cancer tissue of a pancreatic cancer patient (data taken from [2]). Due to the large size of the human genome, a figure of standard dimensions cannot capture a high resolution view of locations of individual mutations along a single axis.

One natural possibility for visualizing mutations at such a broad scale is to make a line plot of mutation frequency for a selected bin size along the genome, i.e. showing the number of mutations in each of 3 Mbp bins along the x-axis (Fig. 1 b), rather than just a binary indication of presence. Such a plot shows how the overall frequency of mutations is distributed across the genome, but does not indicate anything regarding the internal distribution of mutations within each 3 Mpb bin. From such a plot, one has no information of whether the given number of mutations within a 3 Mbp bin are distributed uniformly across the bin, or are mainly restricted to one or more local hotspots, such as kataegis [2] or artifacts [3] within the bin. Indeed, the highly specific (non-uniform) distribution that can be seen in Fig. 1 a represents only 10% of a single bin (300 kbps), and is thus only visible at a resolution much higher than that of a genome-scale line plot.

An RP is an attempt to provide some high-resolution location information along with the global overview of frequency information that is otherwise shown by a line plot. The indication of inter-mutation distance, and the use of a logarithmic scale to demonstrate it, provides a way to visualize hotspots of mutations at a resolution far beyond what is afforded by the resolution provided by the segmentation into 3 Mbp bins along the x-axis.

Figure 1 c shows a genome-scale RP of the pancreatic mutation data. From this RP one can also see that many of the mutations are falling very closely together - mostly at a distance of ∼10−1000 bp between consecutive events, as seen from the values at the y-axis. Still, for a single x-value (typically corresponding to a region of ∼3 Mbps in case of human genome), the RP is only able to visualize the distribution of pairwise distances between consecutive mutations (along with various limitations as discussed in following sections). It is thus not able to provide the full high-resolution view of events as can be seen from a zoomed-in view of locations, as in Fig. 1 a.

Although an RP requires some care and insight in order to make appropriate interpretations, the plot itself can be generated without the need to specify any parameters. In contrast, a line plot of frequency is trivial to interpret, but successful detection of patterns of interest may be highly dependent on the selection of bin size for which the frequencies of mutations are counted.

**Fig. 2:**
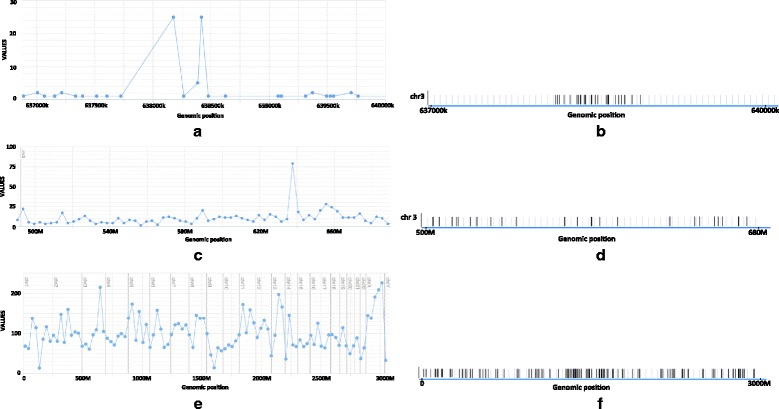
The same kataegis region with different bin sizes. Individual mutation locations within the bin containing a kataegis region for bin size 30Kbps (**a**), 3Mbps (**c**) and 30Mbps (**e**), as well as line plots showing the average density of mutations along those same regions in (**b**), (**d**) and (**f**), respectively. Data taken from [2]

Figure 2 shows how the same kataegis region as discussed above would be represented with different bin sizes in a frequency plot. At small bin sizes, the very high intensity of mutations will cause the kataegis region to stand out clearly. At large bin sizes however (and even despite high mutation intensity), the limited extent of the kataegis region will not contribute sufficiently to the aggregate point count to make the corresponding bin stand out in context of the general variability.

### Interpreting density and frequency of mutations along the genome

A main motivation for the use of RPs is to visualize localized regions of hypermutation. As discussed in the previous section, visualizing such regions in the context of a whole genome is challenging, since their extent may be minuscule relative to the genome size. In order to relate appropriately to mutation density, region extent and total number of mutations, it may be helpful to think of mutation locations in light of a conceptual model, such as a point process.

If mutations were independently and uniformly distributed across the genome, the points could be considered the result of what is called a homogeneous Poisson point process (HPP). An HPP is a stochastic process used in many fields of science as a way of modelling random events along a single dimension that represents a reference variable (generally it is time, in our case it is genome location) [6, 7]. However, the intensity of mutations is not uniformly distributed along the genome, and a generalization of the HPP needs to be considered. The non-homogeneous Poisson point process (NHPP), an extension of a standard HPP, allows the intensity of studied random events (typically referred to as a parameter *λ*) to be a function of the reference variable, e.g. to vary along the genome. The expected distance between mutations follows directly from the intensity of an HPP/NHPP (the expected value for distance is given by 1/ *λ*).

In the absence of full high-resolution information, a natural interpretation is that the locations of individual mutations are distributed without any particular structure within the bounds provided by the visualization setup. The NHPP represents such a natural baseline assumption. The overall intensity varies along the genome, while individual events are assumed to fall uniformly and independently (as in an HPP) within regions of stationary intensity. According to such a baseline, a line plot of frequency in ∼3 Mbp bins along the genome indicates the average intensity (*λ*) for such an NHPP in each bin. Since the frequency only indicates the average intensity (area under the intensity curve) for a given bin, it is based on a heightened frequency value for a particular bin. Therefore it is not possible to distinguish between a small region of high intensity and a larger region of lower intensity (as long as the region of heightened intensity is occurring within a single bin).

In contrast, the inter-event distances provided as y-values on the RP provide a direct indication of intensity level, since the intensity of an HPP directly corresponds to the expected inter-event distances. The presence of several close points (having the same x- and y-values within a limited range) thus indicate one or more (approximately) stationary regions of the corresponding intensity within that bin. In Fig. 1 c, the cluster of points in chromosome 3 at (logarithmic) y-values of around 2 thus indicates the presence of a region of average inter-event distance around 10^2^=100 within the bin corresponding to that particular x-value (corresponding to intensity (*λ*) of 1/100=0.01.

In principle, the extent of such a stationary region of heightened intensity (or total extent in case of multiple regions) could also be approximately derived by looking at the number of distinct points on the plot, but due to issues with congestion (as discussed in a later section), the extent of the heightened region and the total number of mutations within a bin can not be robustly read out.

**Fig. 3:**
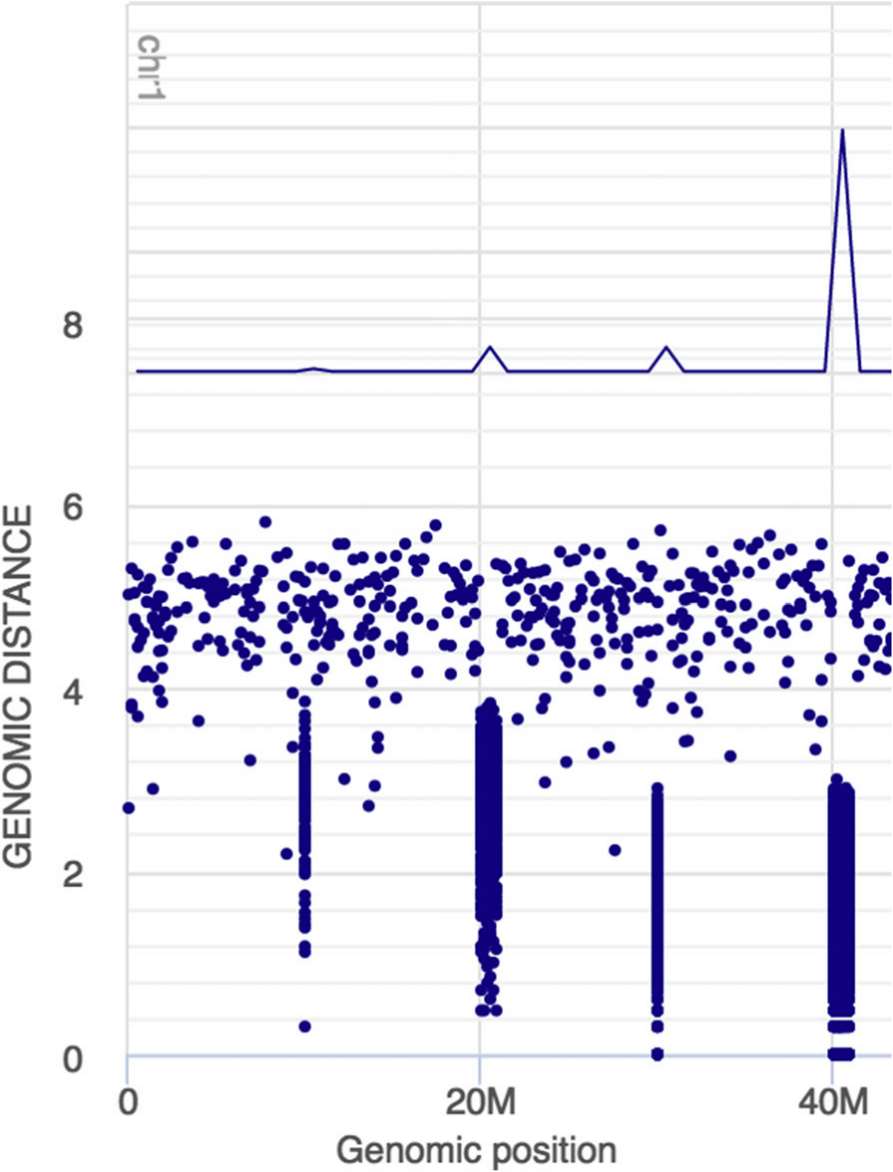
Density and frequency of mutations along the genome. Simulated data with four hotspot regions. The first and second region have the same inter-mutation value, equal to 0.001, while the third and fourth inter-mutational value is in both cases equal to 0.01. The first and third region share the same genomic regions, and so do the second and fourth region

Figure 3 shows a simulated data set containing four hotspot regions (regions of heightened event density), each having a distinct combination of intensity and extent. The line plot here only allows distinction between hotspot regions containing a different number of events, while the RP only allows robust distinction between hotspot regions of different peak intensity.

Table 1 summarizes the plot abilities to distinguish regions based on either peak event intensity or area under the event intensity curve.

**Table 1:** Summary of the frequency line plot and RP abilities to distinguish regions based on either peak event intensity or area under the event intensity curve

	Frequency line plot	Rainfall plot
Intensity (density of mutations in hotspot regions)	No	Directly
Integral (number of mutations in hotspot regions)	Directly	To some degree through point counting
Extent (length of hotspot regions)	No	To some degree by deriving from integral and intensity

Intensity here refers to the average distance between events within a genomic region (density of mutations within a hotspot region). Integral refers to the total number of events within a genomic region (number of mutations within a hotspot region). Extent refers to the length of the genomic region of heightened event frequency (length of hotspot region)

### Detecting recurrent enrichment of mutations at a particular scale

**Fig. 4:**
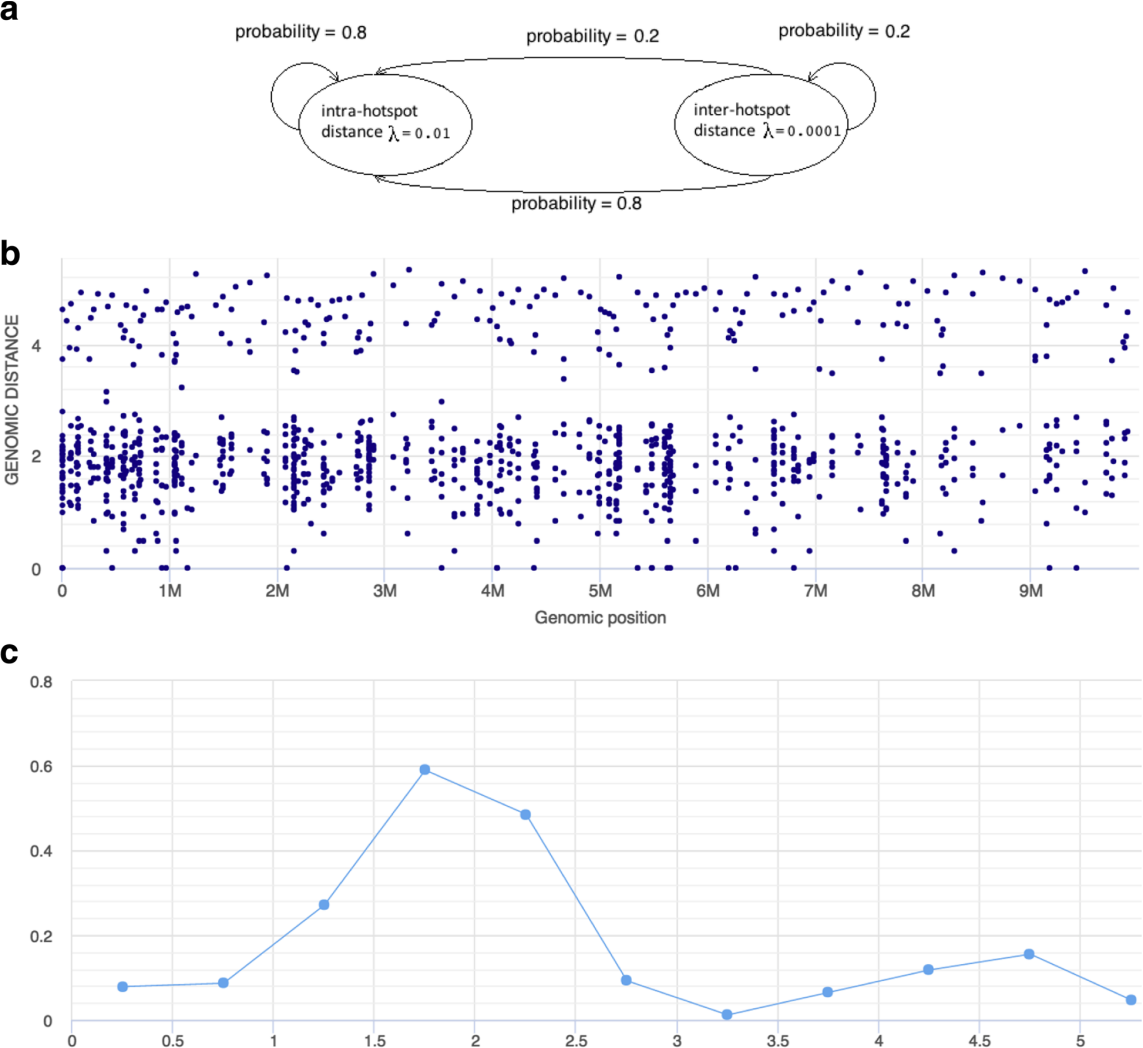
Clustering of mutations as an HMP. HMP consisting of two states (**a**), with a corresponding rainfall pattern that such a process would give rise to (**b**) and a corresponding histogram of inter-event distances (**c**). In this example of a hidden Markov process, there is a high probability (P=0.8) of being in or moving to the state with low intra-hotspot distance (*λ*=0.01), which generates closely spaced events. A more seldomly occurring state (P=0.2) with large inter-hotspot distance (*λ*=0.0001) generates events with large distance to their preceding neighbors. The process defined by these two states generates several distantly spaced hotspots of events, giving rise to two quite distinct horizontal bands in the RP. The same pattern can also be seen as two distinct peaks in the histogram of inter-event distances

In addition to specific exceptional events that give rise to marked local hotspots, there may also be tendencies for SPMs to follow general patterns of varying intensity at specific scales. Such patterns might be associated with biological mechanisms affecting the true distribution of SPMs, or may reflect technical artifacts of sequencing and variant detection. Since RPs use the y-axis to denote inter-mutation distances on a logarithmic scale, mechanisms leading to recurrent enrichment of mutations at a particular scale (clustering) may be spotted as horizontal bands in the plot (enrichment of dots within a restricted range of y-values, across x-values). Figure 4 shows an example of such enrichment at particular inter-mutation distances, based on simulated data.

A challenge in detecting and correctly interpreting such enrichments of particular inter-mutation distances is that visual patterns for a specific data set and region of interest need to be contrasted with what would be a baseline distribution of inter-mutation distances (and with corresponding visual appearance in an RP). What is crucial to note here, is that a uniform and independent distribution of mutations within a region (i.e. a homogeneous/stationary Poisson process) will not result in anything like a uniform distribution of inter-mutation distances (neither on a linear nor on a logarithmic scale), but rather in an exponential distribution with mean value of 1/*λ*. Viewed on a logarithmic scale (as on the y-axis in a RP), such a distribution of distances would show a markedly increased density of points around the y-value corresponding to the mean distance value (1/*λ*), without this denoting any tendency for clustering (recurrent enrichment of mutations at a particular scale). Indications of clustering can thus only be correctly detected as horizontal bands that are not a mere consequence of a general baseline intensity.

To correctly delineate enrichments at specific scales, it may again be useful to consider a conceptual model. By contemplating which patterns of inter-mutation distances are to be expected from such a model, one may more accurately detect the presence of such patterns in real data, as well as connecting the patterns to underlying processes. A hidden Markov process (HMP) may serve as such a conceptual model, as it is capable of producing recurrent distance enrichments and are based on a small number of parameters that are easy to interpret. It would in our setting consist of two or more states: one state where mutations are occurring at a baseline level, and one or more additional states where mutations are occurring at increased intensity due to some particular (biological or technical) mechanism.

Figure 4 shows such a simple HMP consisting of two states (4 a), as well as the rainfall pattern that such a process would give rise to (4 b) and a corresponding histogram of inter-event distances (4 c). Note that such variation in intensity could also be represented by an NHPP with intensity varying between these levels. However, the NHPP would not be bound to the recurrent switching between the same limited set of specific intensity levels and would thus be a less informative model of what is assumed to be an underlying general mechanism. Note also that while a single short region of increased intensity would result in an enrichment of rainfall dots at y-values distinct from the baseline, this would be limited to a single x-value, and thus not form a band in the plot.

### Congestion and saturation in rainfall plots

**Fig. 5:**
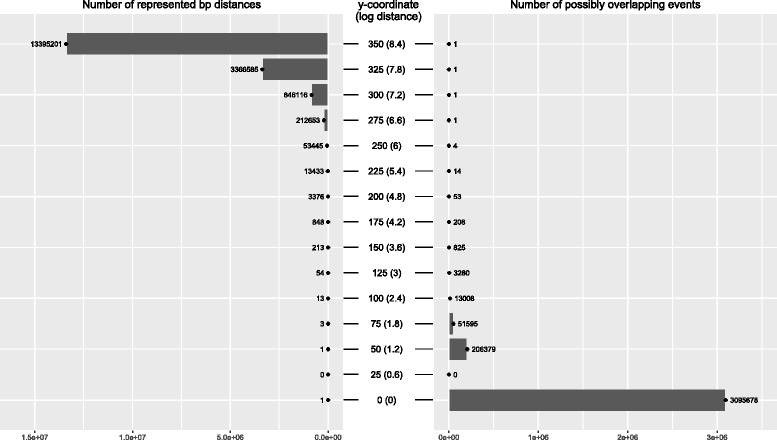
The extent of possible event congestion on a human genome RP with dimensions of 1000*x*351 pixels. The number of distinct represented inter-event distances and the number of possibly overlapping events are displayed for selected RP y-coordinates. RP pixels with low y-coordinates represent few or even no inter-event distances (as seen for y-coordinates 0, 25, 50). At the same time, the distances represented by low y-coordinates are short and allow therefore for a high number of events to share the same x-coordinate. On the other hand, individual pixels with high y-coordinates can represent groups of many distinct inter-event distances (i.e., hundreds, thousands or millions of distances at a time). At the same time, higher y-coordinates represent longer distances, which increasingly limits the number of events that could possibly share the same x-coordinate (with only a single event fitting onto any x-coordinate at the highest y-values). Changing the plot dimensions will influence which distances and which genomic locations will be distinguishable from each other

When illustrating the distribution of events within the human genome, every point on a modestly-sized RP figure (i.e., a standard journal figure) will correspond to a range of genomic locations, as well as a range of inter-event distances, rather than a single genomic location and a single distance value. As a consequence, multiple distinct events may share the same coordinates and overlap on the plot, creating misleading impressions of event density or the lack thereof. Figure 5 shows the possible extent of congestion on a RP of a particular size. The linear scale of the x-axis leads to each x-value uniformly representing a given number of base pairs of the human genome (typically several million bases). The number of inter-event distances represented by a single y-value greatly changes based on the vertical position on the plot, however. As the y-values decrease, individual distances become increasingly easier to distinguish from each other, a feature that is convenient when small distances are of most interest. On the other hand, the congestion is scaled in an inverse manner along the y-axis, with the possibility of event-overlap quickly growing as inter-event distances decrease.

**Fig. 6:**
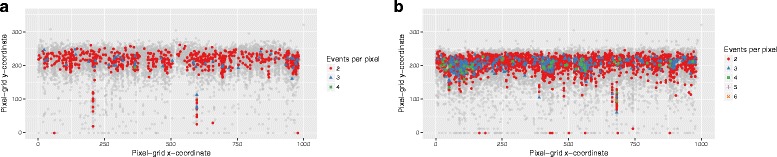
The extent of event-overlap („congestion”) in data. Congestion in the pancreatic cancer data (**a**) and breast cancer data (**b**) used for illustrating kataegis in [2]. The events were projected onto an RP pixel grid with dimensions of 1000*x*351 pixels

Whether congestion represents a problem in practice depends on the nature of the plotted events. In kataegis examples as given by Alexandrov [2], the variety of inter-event distances prevents saturation from being problematic. (Figure 6 a and b show that at most 6 events are ever projected into identical coordinates on 1000*x*351-point RP representations of the original figures. In both cases, enough unique projections remain in order to create apparent „rainfalls”.) However, potentially interesting clusters of events may not be apparent on an RP if only small numbers of events form such clusters (e.g., as few as six events indicating a kataegis region according to [2], which would correspond to 5 plotted distance-values with possible overlaps). In addition, several independent event clusters might appear as a single cluster if they fall within a single x-value on the RP. In general, the standard RP is not suitable for illustrating situations in which the events of interest are expected to appear close to each other and at distance intervals with little or no diversity, e.g. recurring mutations in a group of patients.

**Fig. 7:**
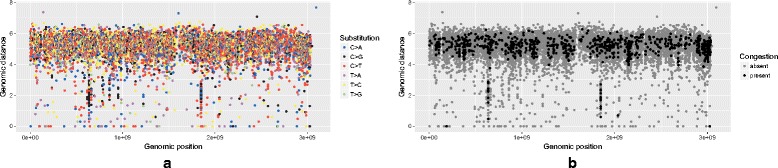
Pancreatic cancer variants. Variant are plotted in an order based on their genomic location (**a**) and projected on a 1000*x*351 pixel RP grid with higlighted sites of congestions (**b**)

**Fig. 8:**
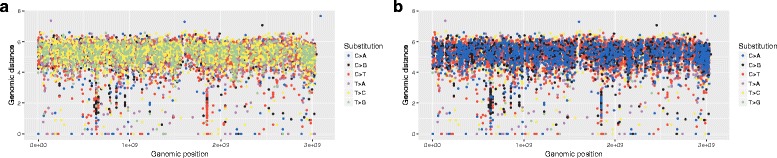
Pancreatic cancer variants. Variants are plotted in an order based on the substitution type, with either *C*>*A* variants were plotted first (**a**) or last (**b**). Although based on the same data and using a shared color scheme, the two plots give very different impressions of which type of mutation is the most prevalent one

Plotting every event irrespective of the others seems to be the standard approach when creating an RP. Not taking the congestion into account has an additional side-effect if subsets of the plotted data are assigned different colors: the order in which the individual events are plotted becomes important. Figures 7 and 8 show the same pancreatic cancer data as used in [2]. In Fig. 7 a, the variants were plotted in an order based on their genomic location, while Fig. 7 b highlights sites of congestion in an RP with dimensions of 1000*x*351 pixels. In Fig. 8 a and b, the variants were plotted in an order based on the substitution type (8 a follows the order in which the substitution types are listed in the legend, with *C*>*A* variants plotted first and *T*>*G* variants last; while Fig. 8 b follows a reversed order, with *T*>*G* variants plotted first and *C*>*A* variants last). Interestingly, 8 a seems to be the order used in [2], even though this ordering creates a misleading impression of some substitutions being more common than others by obscuring the events that were plotted first (besides congestion as described above, the size of the points on a plot can contribute to this problem).

One way to alleviate plot congestion could be through zooming, i.e. making an RP of the same resolution for a smaller part of the genome. Such zooming may be offered interactively, or based on manually re-creating a plot for a specified subpart of the genome. It can certainly be useful to make plots for each chromosome separately, or even for very small genomic regions of particular interest. However, part of the strength of the RP is its ability to convey patterns across multiple scales in a single overview plot. Reliance on multiple zoomed-in views does not allow this same degree of summarization or possibility for direct visual contrasting of patterns seen throughout the genome.

### Guidelines for using and interpreting rainfall plots

As described in the previous sections, the RP is able to show a variety of information related to the distribution of mutations along the full scale of a genome. In order to recognize this information, it is crucial to read the plot in a precise manner (and not merely rely on intuition), beeing aware of caveats of the plot that could potentially distort the presented information. The following is a simplified guide to creating and interpreting RPs (all explicit values are based on the human genome):

1. Intuitively, a low-intensity region will be seen as a thinly populated band of dots in the upper part of the plot (since low density implies high average inter-mutation distance), while a high intensity region (mutation hotspot) will be seen as a dense collection of points lower in the plot („rainfall”). Remember that since the x-axis spans a huge number of individual values (∼3 billion bases), a single x-value represents many megabases, and mutations plotted proximally along the x-axis may thus still be megabases apart. Also remember that while the y-value shows the distance to the previous mutation along the genome, this previous mutation needs not to be located closely in the plot (since it can have a very different y-value).
2. To read out the intensity of a given region more precisely, consult the y-axis to get an impression of the typical inter-mutation distance. Remember that since the y-axis is logarithmic, the middle y-value of a set of points does not represent the average inter-mutation distance of these points (mind the difference between the average on the underlying linear scale and the logarithmic scale of the plot).
3. To get an impression of the number of mutations in a given region, consider the number of distinct dots in the plot. Remember that there can be congestion in the plot, meaning that multiple mutations are assigned the same (*x,y*)-value and thus are represented by a single dot. Due to stochasticity, this is not likely to be the case as long as a part of the plot in question is not close to being saturated (if there is more unoccupied space than dots in a region of interest in the plot).
4. When multiple datasets are represented by unique colors in the same plot, (*x,y*)-values associated with more than one dataset should be marked by a neutral color (e.g. black), rather than being arbitrarily assigned the color corresponding to the dataset that is plotted last. If using an existing rainfall plotting functionality that violates this recommendation, be cautious in concluding about which datasets (colors) are the more prevalent (in a particular region or in the genome as a whole). Preferably, create the plot again after permuting some of the characteristics of the data (e.g., variant order or types) to see if some aspects of the plot unexpectedly change.
5. In case there is any strong recurrent enrichment of mutations at a particular scale, this would show as a horizontal band of dots that comes as an addition to a main distribution of dots (at a separate level of y-value). The scale at which such recurrence occurs could be read out as the rough y-value at which the band is positioned.

## Conclusions

The RP is a curiously defined plot that has recently gained a lot of popularity for visualizing the distribution of mutations across a large genome. It combines a global indication of relative genome location (x-axis) with a local indication of density (y-axis). The RP may appear to simply be an exotic and inefficient visualization of frequency (which could be conveyed more efficiently through a standard line plot). However, a careful analysis shows that its use of a logarithmic y-axis to display inter-mutation distances allows the plot to e.g. capture very short high-intensity regions that would not be detectable in a binned frequency plot.

At the same time, the RP has certain weaknesses. Something as basic as the number of mutations within a given region (which can be read off directly at the y-axis of a standard frequency plot) is from the RP only indicated indirectly and imprecisely (evaluating frequency requires counting of dots, and even this is not necessarily precise due to potential congestion issues).

The advantage of a visual approach, like the creation of an RP, is that a broad range of patterns may be detected and communicated. The RP is thus well suited for explorative analyses. When searching for a limited set of patterns with well-defined formal representations, automatized detection approaches will in general be preferable. In conclusion, the RP allows patterns across a broad range of scales to be detected visually, without the need for any parameterization. At the same time, a deep understanding of the plot is needed in order to read the contained information precisely and for appreciating its potentially misleading aspects.

## Methods

We here provide in the first subsection a formal definition of the stochastic processes, i.e. HPP and NHPP, which are used in the paper as conceptual models of the distribution of mutations along the genome. Further mathematical details can be found in [6]. In a second subsection, we describe tools which can be used to reproduce the majority of results presented in the paper.

### Stochastic process

A stochastic process *x*={*X*(*t*),*t*∈*T*} is defined as a collection of random variables [6]. Let us denote with *t* the time and with *X*(*t*) the state of the process *x* at time $t, t \in \mathbb {N}$, for each *t* in the time set *T*, *X*(*t*) is a random variable.

The process *x* is said to be a discrete-time stochastic process and a continuous-time stochastic process if the index set *T* is a countable set and a continuum set respectively.

Let {*N*(*t*),*t*∈*T*} be a discrete-time stochastic process (counting process), representing the number of events *N*(*t*) that have occurred up to time t. The counting process {*N*(*t*),*t*≥0} must satisfy the following conditions [6]:

- $N(t) \geq 0, t \in \mathbb {N}$,
- $N(t) \in \mathbb {N}$,
- if *s*<*t* then *N*(*s*)≤*N*(*t*).

If *s*<*t* then *N*(*t*)−*N*(*s*) is the number of events that have occurred in the interval (*s,t*].

The counting process has independent and stationary increments if the numbers of events that occur in two non-overlapping intervals are independent i.e. *N*(*t*_1_),*N*(*t*_2_)−*N*(*t*_1_),… for *t*_1_<*t*_2_…<*t*_*n*_ and the distribution of the number of events that occur in any interval of time depends only on the length of the time interval; the number of events in the interval (*t*_1_+*s,t*_2_+*s*], that is *N*(*t*_2_+*s*)−*N*(*t*_1_+*s*), has the same distribution as the number of events in the interval (*t*_1_,*t*_2_], that is *N*(*t*_2_)−*N*(*t*_1_) [6].

#### Homogeneous poisson process

The homogeneous Poisson process [6, 7] is defined as a counting process {*N*(*t*),*t*≥0} with rate *λ*, *λ*>0, if the following conditions are satisfied:

- *N*(0)=0,
- the process has independent increments,
- the number of events in any interval of length t is Poisson distributed with mean *λ* *t*, $P \{ N(t + s) - N(s) = n \} = e^{\lambda t} \frac {(\lambda t)^{n}}{n!}, n = 0, 1 \ldots $ for *s,t*≥0. The Poisson process has stationary increments and *E*[*N*(*t*)]=*λ* *t*.

#### Non-homogeneous poisson process

A non-homogeneous Poisson process (NHPP) is a generalization of the HPP, where the rate parameter *λ* is not constant, but is a function of time *t*, *λ*(*t*). Let {*N*(*t*),*t*≥0} be a counting process representing the cumulative number of mutations occurred in the interval (0,*t*]. Then the expected value of mutations *N*(*t*) is defined by *λ*(*t*), which is called a mean value function of the NHPP. The model can be formulated as follows [8, 9]:

$$\begin{array}{*{20}l} P \{ N(t) = n \} = e^{\lambda (t)} \frac{(\lambda (t))^{n}}{n!}, n = 0, 1 \ldots \text{for}\; t \geq 0 \end{array} $$

Given the above NHPP, let *X*_1_ denote the time when the first mutation occurs, let *X*_2_ be the time between the first and second mutation, then for *n*≥1, let *X*_*n*_ be the time between the (*n*−1)st and *n*th mutation. Then, the sequence of random variables *X*_1_,*X*_2_,…,*X*_*n*_ represents the inter-arrival times between mutations and each random variable is independent from each other and follows an exponential distribution with mean 1/*λ*(*t*).

### Tools

Figures 1 a, 2 b, d and f were generated using the UCSC Genome Browser. Figures 1 b, c, 2 a, c, e, 3 and 4 b were generated using the webtool „Create a dynamic rainfall plot with corresponding frequency plot” in The Genomic Hyperbrowser (GHB) [10]. Data for Fig. 3 was calculated using the tool „Generate synthetic datasets with Poisson distribution” in GHB. Other data were taken from [2] (direct link: ftp://ftp.sanger.ac.uk/pub/cancer/AlexandrovEtAl/) for pancreatic cancer patient APGI _1992 and breast cancer patient PD6043a and transform into ’bed’ format file using tool „Convert data from paper [2] into bed file”. Figure 4 c was plotted using the tool „Create event distribution density” in GHB. Figure 5 can be reproduce using tool „Reproduce figure with extent of possible event congestion”. Tool „Generate a static rainfall plot with per-pixel event-counts” is used to create Fig. 6. Figures 7 and 8 were generated using tool „Generate a static rainfall plot”.

All plots and the information necessary for their reproduction can be found at https://hyperbrowser.uio.no/rainfall. Plots generated by GHB webtools can be reproduced using the redo-functionality provided by the underlying Galaxy system. Plots generated in R are accompanied by their respective R code and the data files used to generate them. Plots generated by the UCSC Genome Browser are accompanied by URLs and form inputs required to generate similar plots in the current version of UCSC.

## Additional file

Additional file 1 Supplementary material. The file includes two definitions. The first defines how to formally provide a rainfall plot for the whole genome. The second defines how to discretize a whole genome rainfall plot (how to formally transform values in order to fit a grid). (PDF 32 kb)

## Abbreviations

GHB: The Genomic Hyperbrowser
HMP: hidden Markov process
HPP: Homogeneous Poisson point process
NHPP: Non-homogeneous Poisson point process
RP: Rainfall plot
SPMs: somatic point mutations
