# Supplementary material for: The rainfall plot: its motivation, characteristics and pitfalls
Source: BMC Bioinformatics. 2017 May 18;18:264. doi: 10.1186/s12859-017-1679-8 (PMC5437519; doi:10.1186/s12859-017-1679-8)
Supplement: Supplementary file 1 — Supplementary material. The file includes two definitions. The first defines how to formally provide a rainfall plot for the whole genome. The second defines how to discretize a whole genome rainfall plot (how to formally transform values in order to fit a grid). (PDF 32 kb) [file 12859_2017_1679_MOESM1_ESM.pdf]

1 Supplementary information

1.1 Definition of rainfall plot for the whole genome

A point mutation event (c, p) is represented by a chromosome identifier c (e.g., „chr1") and an integer p that specifies a base pair position on the chromosome.

Let $c_j, j \in 1, \ldots, M$ be the contigs/chromosomes selected for plotting (further collectively refered to as the „whole genome" for simplicity). The chosen ordering (e.g., „chr1", „chr2", …, „chr21", „chr22", „chrX", „chrY") is of consequence, as it will determine the ordering of chromosomes on the x-axis of the rainfall plot. Let $S = \{(x_i^j, y_i^j), i \in \{1, 2, \ldots, N^j - 1\}, j \in \{1, 2, \ldots, M\}\}$ be a set of points created chromosome-wise according to definition 2.1 but encompassing the whole genome.

To avoid chromosome overlaps while creating a rainfall plot based on points from S, the values representing event base pair position for each chromosome need to be increased (offset) by the summed length of all the preceding chromosomes, creating a point set S^*. Scatterplot based on points from S^* will thus have x-values in the range $[0, \ldots, genome\_size]$.

Let s_j for $j \in \{1, \ldots, M\}$ be the lengths of the individual chromosomes. Then let s_j^* be the chromosome-specific base-pair increments (offsets) defined as follows:

$$
\begin{aligned}
&s_1^* = 0, \\
&s_j^* = s_{j-1}^* + s_{j-1} \quad \text{for} \quad j \in 2, 3, \ldots, M.
\end{aligned}
$$

For each chromosome $j, j \in \{1, \ldots, M\}$, the s_j^* offset will be applied to all of its elements (x_i^j, y_i^j) for $i \in \{1, \ldots, N^j - 1\}$ in the following way:

$$
(x_i^{j*}, y_i^j) = (s_j^* + x_i, y_i^j).
$$

The final point set could then be defined as $S^* = \{(x_i^{j*}, y_i^j), i \in \{1, 2, \ldots, N^j - 1\}, j \in \{1, 2, \ldots, M\}\}$

To simplify the notation, the indexing can be changed from $(x_i^{j*}, y_i^j), i \in \{1, \ldots, N^j - 1\}, j \in 1, \ldots, M$ to $(x_k, y_k), i \in \{1, \ldots, K\}, K = \sum_{j=1}^{M} N^j - 1$.

1.2 Transformation of the rainfall plot for the whole genome in order to fit a grid

A rainfall plot for a whole genome of total size K and with its largest chromosome being of length C can be further standardized (ensuring constant value ranges for both axes on any rainfall plot for the given genome) and transformed in order to fit a grid of size (W, H):

$$
\begin{aligned}
&x_k^{TS} = \lceil \frac{W * x_k}{K} \rceil, \\
&y_k^{TS} = \lceil \frac{(H - 1) * y_k}{log(C - 1)} \rceil.
\end{aligned}
$$
